# Supplementary figures and images for: Sugar- and Artificially Sweetened Beverages Consumption Linked to Type 2 Diabetes, Cardiovascular Diseases, and All-Cause Mortality: A Systematic Review and Dose-Response Meta-Analysis of Prospective Cohort Studies
Source: Nutrients. 2021 Jul 30;13(8):2636. doi: 10.3390/nu13082636 (PMC8402166; doi:10.3390/nu13082636)

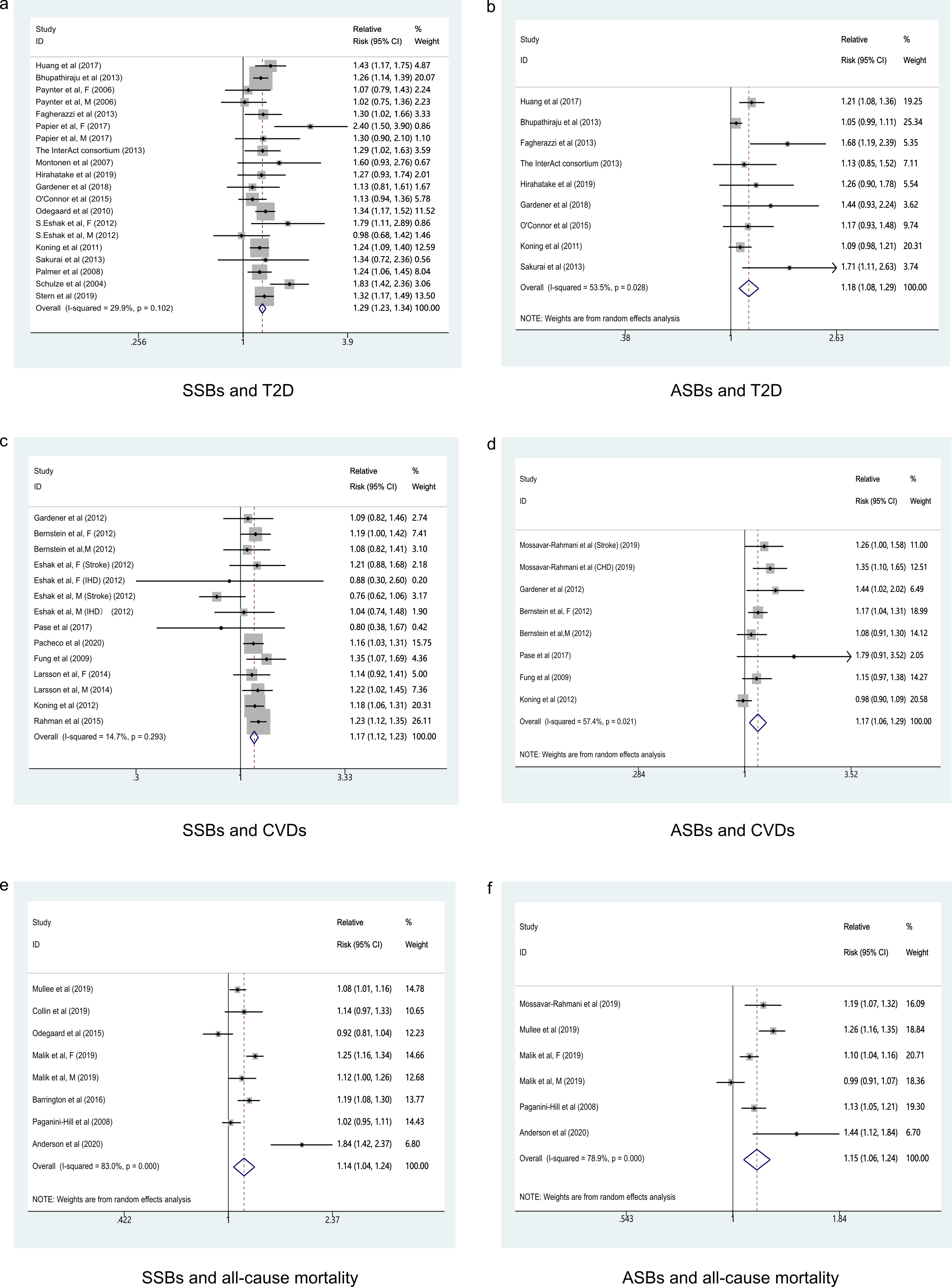

Supplement: Supplementary file 1 [file nutrients-13-02636-s001.zip › figure 2.png]

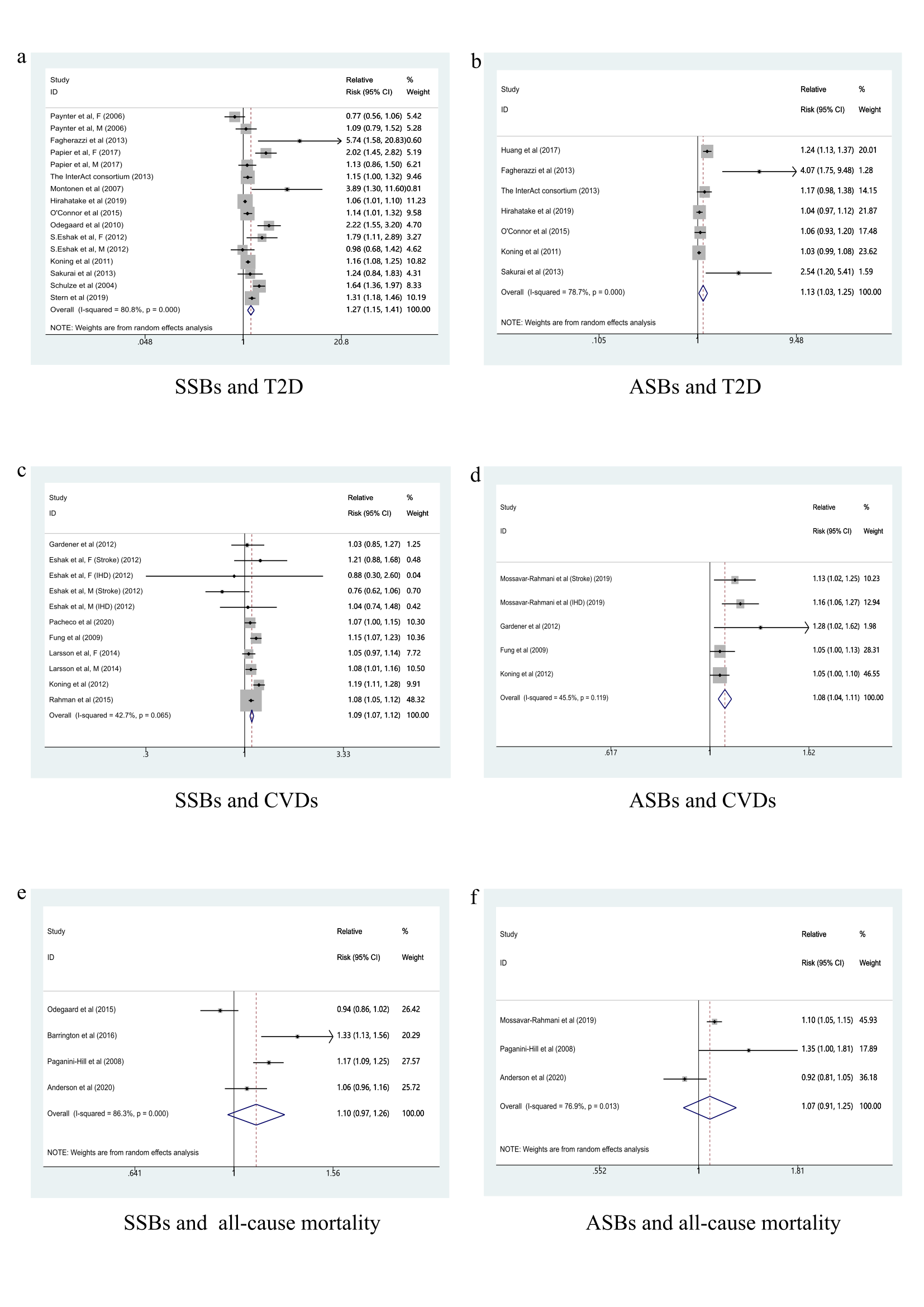

Supplement: Supplementary file 1 [file nutrients-13-02636-s001.zip › figure4.png]
